# The prevalence and distribution of *Acidobacteriota* in the Nama Karoo of South Africa

**DOI:** 10.3389/frmbi.2026.1653994

**Published:** 2026-02-13

**Authors:** Janca Pieters, Tersia Andrea Conradie, Karin Jacobs

**Affiliations:** Department of Microbiology, Stellenbosch University, Stellenbosch Central, Stellenbosch, Western Cape, South Africa

**Keywords:** abiotic properties, *Acidobacteriota*, *Acidobacteriota* subdivisions, arid soils, co-occurrence, relative abundance

## Abstract

The phylum *Acidobacteriota* is ubiquitous and a dominant bacterial group in arid lands, playing a crucial role in nutrient cycling and ecosystem functioning. This study explores *Acidobacteriota* in Southern African arid lands through two complementary approaches. A meta-analysis of 240 soil samples revealed relative abundances ranging from 0.008% to 39.1%, with pH identified as the primary driver of community variance. In addition, 96 bulk soil samples from the Nama Karoo were analyzed using full-length 16S rRNA gene sequencing (V1–V9). *Acidobacteriota* abundance ranged from 2.3% to 12.2%, with Subdivisions 3, 4, and 6 being the most dominant, while rare subdivisions, such as 2 and 9, showed location-specific distributions. Significant beta-diversity differences (p = 0.002) were linked to soil moisture, electrical conductivity, and nitrate availability, and some subdivisions exhibited correlations with organic carbon and nitrate. Co-occurrence patterns with *Planctomycetota* and *Armatimonadota* suggest potential biofilm formation and shared ecological niches. This study provides the first comprehensive assessment of *Acidobacteriota* in Southern African arid lands, highlighting dominant and rare subdivisions, localized ecological associations, and the need for future work on their metabolic functions and adaptive strategies in arid ecosystems.

## 1 Introduction

Arid lands are characterized by extreme temperatures, limited water availability, and elevated levels of ultraviolet radiation and represent unique environments that are increasingly becoming a focus of ecological and microbial research (Lancaster et al., 1984; Laity, 2009; Zomer et al., 2022). These regions, which make up about one-third of the Earth’s terrestrial surface, are distinguished from temporary drought conditions by their permanent aridity, often quantified by the Aridity Index (Peel et al., 2007; Pointing and Belnap, 2012; Liang et al., 2022). Within Africa, arid lands constitute approximately 45% of the continent’s surface and host biodiversity hotspots such as the Horn of Africa and the Succulent Karoo, both known for their plant diversity and endemism (Pimm et al., 2014; Myers et al., 2000; Zhang et al., 2023). The Nama Karoo is South Africa’s third-largest biome, accounting for around 20% of the country’s total area, and harbors more than 2–000 plant species, nearly 800 (18%) of which are endemic. Climate change, driven by rising temperatures and shifting precipitation patterns, significantly alters soil properties such as pH, aggregate stability, and cation exchange capacity, with cascading effects on ecosystem functioning (Maurer et al., 2020; Deng et al., 2022; Shi et al., 2022; Li et al., 2024). In these arid ecosystems, the stability and productivity of plant and faunal communities are tightly coupled to the activity of soil microbial communities, which regulate nutrient turnover, organic matter decomposition, and other key biogeochemical processes (Bardgett and Van der Putten, 2014; de Menezes et al., 2017; Ramond et al., 2022; Chhiba et al., 2023). While much research has focused on the biodiversity of flora and fauna in these regions, microbial communities particularly soil bacteria are essential yet understudied components of arid ecosystems (Maestre et al., 2015).

*Acidobacteriota*, a bacterial phylum widespread in soils, play crucial roles in biogeochemical cycles, including carbon and nitrogen cycling (Chaudhary et al., 2019; Kielak et al., 2016). The phylum *Acidobacteriota* is taxonomically divided into a series of phylogenetically distinct lineages, commonly referred to as subdivisions (SDs), and the current classification system recognizes 15 class-level units, 6 of which contain cultured representatives: *Terriglobia* (SD 1 and 3), *Blastocatellia* (SD 4), *Holophagae* (SD 8), *Vicinamibacteria* (SD 6), *Candidatus* Polarisedimenticolia (SD 22), and *Thermoanaerobaculia* (SD 25) (Dedysh and Sinninghe Damsté, 2018; Dedysh and Yilmaz, 2018). These bacteria are often found in oligotrophic and acidic environments, which suggests they could be particularly important in arid lands where soil resources are limited (Scola et al., 2018; Bereczki et al., 2024). Genomic and physiological studies have revealed that *Acidobacteriota* possess a remarkable metabolic versatility that enables them to thrive under resource-limited conditions. Members of this phylum encode a wide variety of carbohydrate-active enzymes involved in the utilization, degradation, and biosynthesis of carbohydrates, allowing them to utilize diverse carbon sources such as glucose, xylose, cellobiose, arabinose, mannose, rhamnose, starch, and lactose (Ward et al., 2009; Belova et al., 2018; Eichorst et al., 2018; de Chaves et al., 2019; Costa et al., 2020; Kristensen et al., 2021). This capacity highlights their important role in soil carbon turnover and organic matter decomposition, particularly in nutrient-poor soils. Furthermore, *Acidobacteriota* also contribute significantly to nitrogen cycling. They possess genes for nitrate, nitrite, and nitric oxide reduction, as well as ammonium assimilation, indicating their involvement in both assimilatory and dissimilatory nitrogen pathways (Eichorst et al., 2018; Kalam et al., 2020; Rajeev et al., 2015). For example, *Aridibacter nitratireducens*, isolated from soil, can utilize nitrate as an electron acceptor and metabolize a variety of organic substrates, reflecting adaptability to nitrogen-limited environments. Additionally, several members possess high-affinity hydrogenases, enabling them to scavenge atmospheric hydrogen, potentially supporting survival under nutrient-depleted conditions such as arid regions (Greening et al., 2015; Myers and King, 2016). They can also respire oxygen across a range of concentrations through low- and high-affinity terminal oxidases, which may allow adaptation to fluctuating oxygen availability in soils (Eichorst et al., 2018; Kalam et al., 2020).

Despite their broad metabolic potential and ecological significance, little research has been conducted on the distribution and functional roles of *Acidobacteriota* in arid lands of South Africa. Understanding these roles is essential for elucidating the contribution of *Acidobacteriota* to ecosystem processes in these fragile environments.

Previous studies indicate that *Acidobacteriota* thrives in soils across various ecosystems, including forests (relative abundance ~7.4% to 31.1%), peatlands (relative abundance ~3% to 66%), and agricultural soils (relative abundance ~14.6% to 28.0%), often in low pH and nutrient-poor conditions (Jones et al., 2009; Lauber et al., 2009; Navarrete et al., 2013; Chaudhary et al., 2019; Nan et al., 2022; Golovchenko et al., 2023). Their presence in arid lands has been documented in global studies, where the relative abundance can range between ~ 14% to 28% (Maisnam et al., 2023). A study by Maestre et al. (2015), revealed that bacterial communities in arid regions are dominated by *Acidobacteriota*, along with *Pseudomonadota* and *Actinomycetota*. A study by Nan et al. (2022) on bacterial communities in the soil of arid regions in Northwest China found that *Acidobacteriota* was one of the most abundant groups, followed by *Pseudomonadota* and *Bacteroidota*.

Given that a substantial portion of South Africa is classified as arid lands, understanding the distribution and role of *Acidobacteriota* in these soils is crucial for biodiversity conservation and ecosystem management (Monadjem et al., 2018). The aim of this study is to provide the first comprehensive assessment of *Acidobacteriota* subdivisions (SDs) in Southern Africa’s arid lands by combining a meta-analysis of publicly available datasets with new data generated from the Nama Karoo, South Africa, the first time this region has been examined in this context. Soil samples were analyzed using Oxford Nanopore sequencing, and the integration of secondary data was used to corroborate and strengthen the interpretation of the newly generated dataset. Together, this approach provides a more robust understanding of *Acidobacteriota* diversity and ecological significance in arid soils, while underscoring the need for further research into their metabolic functions and adaptive strategies.

## 2 Materials and methods

### 2.1 Acquisition of secondary *Acidobacteriota* data

To gain a preliminary understanding of *Acidobacteriota* in arid lands of Southern Africa, a meta-analysis was conducted. All publicly available data from peer-reviewed journals (published between January 2022 and June 2024) focusing on microbial community analysis in arid lands of Southern Africa, along with their soil chemical properties, were downloaded from GenBank. All sampling locations of secondary data are depicted in **Supplementary Figure S1**. A total of 300 samples of 16S rRNA sequence data and all available physicochemical data related to soil were analyzed. After quality assessment, diversity data was analyzed using MOTHUR (Version 1.48.0; Schloss et al., 2009; Winand et al., 2019; Akaçin et al., 2023). Quality filtering was applied to remove primers, sequences with ambiguous bases, homopolymers longer than 8 bp, sequences outside the expected size range, and those with a quality score below 25. Chimeric sequences were identified and removed using VSEARCH (Version 2.16.0; Rognes et al., 2016). The filtered sequences were clustered into operational taxonomic units (OTUs) and classified using two reference databases: SILVA (Version1.3, http://www.arb-silva.de) and EzBioCloud (https://www.ezbiocloud.net) with a cut-off value of 80% (Quast et al., 2013; Yoon et al., 2017; Chalita et al., 2024). Sequences classified as unknown, eukaryotes, mitochondria, or chloroplasts were excluded from further analysis.

### 2.2 Nama Karoo

#### 2.2.1 Study sites and collection of soil samples

**Figure 1:**
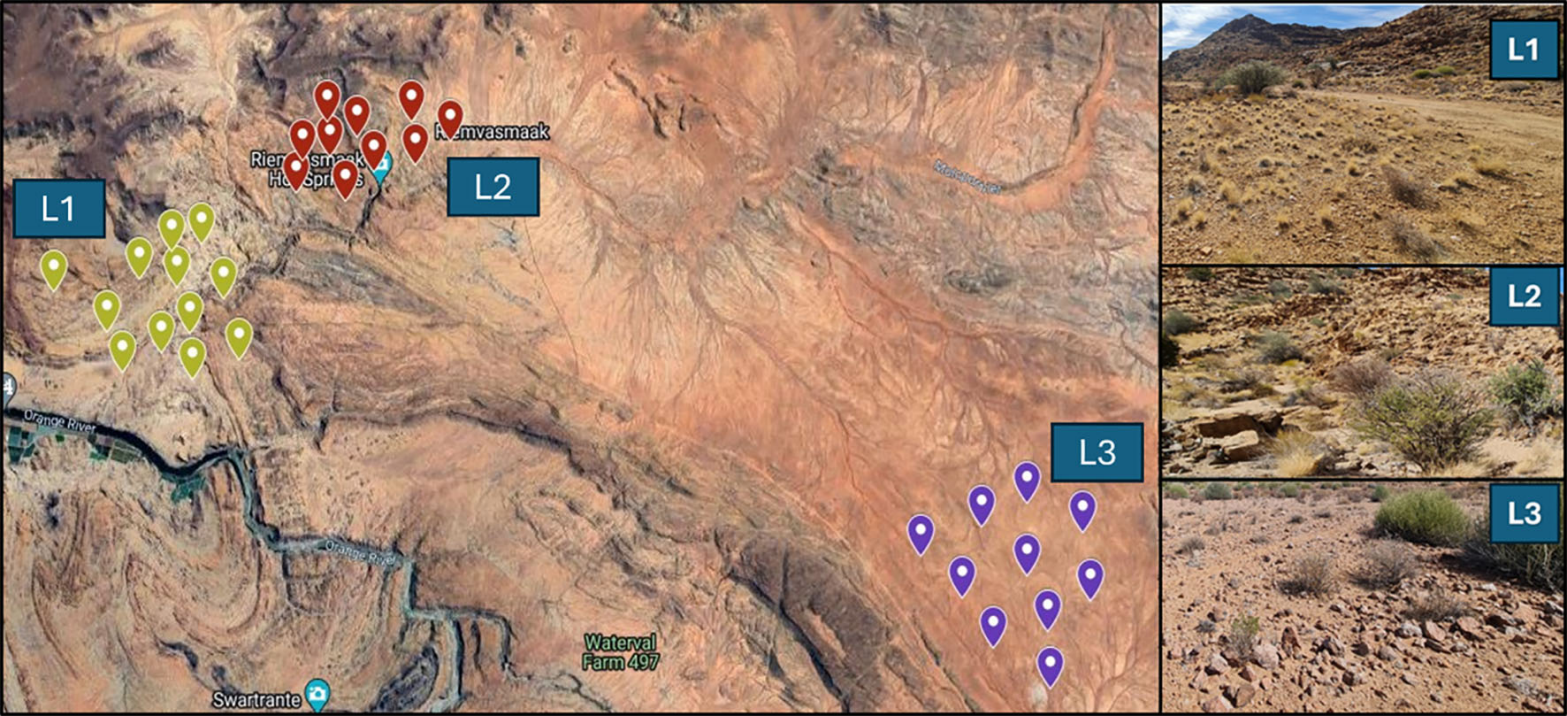
Sampling locations in the Nama Karoo of South Africa (Location 1 n= 12; Location 2 n =10; Location 3 n= 10).

Three sampling locations were selected within a 50 km radius in the Nama Karoo, South Africa, during August 2023: Location 1 [L1; 29°21’53.7”S, 18°28’01.8”E], Location 2 [L2; 28°27’49.7”S, 20°17’02.3”E], and Location 3 [L3; 28°38’51.4”S, 20°26’48.5”E]. These regions have a mean annual precipitation between 127 mm and 254 mm with temperatures ranging from 2 °C to 40 °C (**Figure 1**). A total of 96 bulk soil samples were collected. Briefly, at each location, random plots of 1 m × 1 m were identified and three soil subsamples of approximately 150 g each were taken randomly within the first 10 cm of the soil surface. All samples were collected away from plants to avoid roots. Where present, the top layer of organic material was removed before soil collection (Keet et al., 2019). The three soil subsamples were combined and homogenized to form a composite sample.

#### 2.2.2 Analysis of soil abiotic properties

Soil moisture, electrical conductivity (EC), and pH were measured as outlined by Anderson and Ingram (1994). Soil pH was determined in a soil slurry in ddH_2_O (1:5) solution using an Ohaus Starter 2100 bench pH meter (Ohaus, Switzerland), and EC was measured using an Aqualytic AL15 electrochemistry meter (Lab Unlimited, Ireland). Soil moisture was calculated by weighing 10 g of soil into a sterile falcon tube, desiccating at 37 °C for 7 days, and recording post-incubation weight loss per gram of dry soil. All absorbance readings of downstream tests were read on an iMark Microplate Absorbance Reader (Bio-Rad, USA) and 0.1 g of soil was used unless stated otherwise.

To determine nitrogen mineralization ammonium concentrations in soil were measured at day 0 and day 7. All required reagents were prepared following the protocol outlined by Anderson and Ingram (1994). Absorbance at 655 nm was recorded for day 0 samples, and net nitrogen mineralization was calculated based on the change in ammonium concentration after 7 days of incubation at 37 °C. Nitrate levels were measured using a salicylic acid colorimetric method. Soil extracts were mixed with 5% salicylic acid in sulfuric acid and left for 30 minutes. Sodium hydroxide (4 M) was then added, and the samples were incubated for 1 hour to allow full-color development and absorbance was measured at 410 nm. Available phosphorus was determined with Bray II solution and organic carbon with Walkley-Black method (Bray and Kurtz, 1945; Walkley, 1947; Anderson and Ingram, 1994).

Active carbon, a labile fraction of soil organic carbon, was measured using the potassium permanganate (KMnO_4_) oxidation method (Weil et al., 2003). Soil samples were treated with 0.2 M KMnO_4_ in 1 M calcium chloride (CaCl_2_) solution, and the reaction was conducted in the dark for 10 minutes to oxidize easily oxidizable organic matter. After settling, 20 µL of the supernatant was transferred to a new tube with 1.98 mL ddH_2_O water. The absorbance of the diluted solution was measured at 550 nm.

#### 2.2.3 Soil enzyme assays analysis

Soil enzyme activities measured included: β-glucosidase, urease, and alkaline-acid phosphatase (Dick et al., 1997). Assays were performed with a total of 0.1 g of soil, in triplicate. For β-glucosidase activity, p-nitrophenyl-β-D-glucopyranoside (PNG) was used as the substrate. Samples were incubated with the substrate and buffer solution (THAM 12.1 g/L, Maleic acid 11.6 g/L, Boric acid 6.3 g/L, Citric acid 14.0 g/L, NaOH 19.52 g) at pH 6.0 at 37 °C for 1 hour. After incubation, 0.5 M CaCl_2_ and THAM buffer (pH 12) were added, and absorbance was measured at 410 nm. Urease activity was determined by incubating soil samples in a urea solution (720 mM) with borate buffer (pH 10) at 37 °C for 2 hours. After incubation, a KCl–HCl solution was added, and the mixture was incubated for an additional 30 minutes at room temperature and absorbance measured (660 nm). Both acid and alkaline phosphatase activities were measured using p-nitrophenyl phosphate as the substrate at pH 6.5 and 11, respectively. Samples were incubated at 37 °C for 1 hour, and p-nitrophenyl was extracted using 0.5 M CaCl_2_ and 0.5 M NaOH, with absorbance measured at 410 nm.

The fluorescein diacetate (FDA) hydrolysis assay, was used as a proxy of total microbial activity, following the method described by Green et al. (2006). A total of 0.5 g of soil was combined with 12.5 mL of 1× PBS buffer (pH 7.4) and 0.25 mL of 4.9 mM FDA dissolved in acetone and incubated at 43 °C for 2 hours under constant agitation. After incubation, FDA hydrolysis was halted by adding 40 μL of acetone to 1 mL of soil slurry. Samples were then centrifuged at 8800 g for 5 minutes, and fluorescence was measured (490 nm).

### 2.3 Bacterial community structure analysis

#### 2.3.1 DNA extraction and 16S ribosomal RNA gene PCR amplification

DNA of soil samples were extracted using the ZR *Quick*-DNA Fecal/Soil Microbe Kits (Zymo Research, USA) according to the manufacturer’s instructions with the following modifications to increase final DNA concentration; 0.1% (W/V) of Polyvinylpyrrolidone (PVP) was added to genomic lysis buffer and DNA Elution Buffer was heated at 65 °C for 5 minutes on a heating block (Omega Scientific, USA) (Gray and Herwig, 1996). PCR amplification of successfully extracted genomic DNA was performed with primers specific to the V1-V9 region of the 16S rRNA gene (Dos Santos et al., 2019). The total reaction volume (25 μL) contained 5 µL of 1x KAPA Taq Hotstart Buffer, 2 µL of 1.5 mM MgCl_2_, 0.5 μL dNTP mix, 075 μM forward [27F (5’-ACTCCTACGGGAGGCAGCAG-3’)] and reverse primers [1492R (5’-GGTTACCTTGTTACGAGTT-3’)], 0.2 µL of 0.5 U KAPA Taq HotStart DNA Polymerase, and 2 μL of template DNA (Dos Santos et al., 2019). Products were amplified using the 2720 Thermal Cycler (Applied Biosystems, USA) with following settings: Initial denaturing at 95 °C for 5 minutes, followed by 35 cycles of 95 °C for 30 seconds, 58 °C for 30 seconds and 72 °C for 1 minute. A final extension was completed at 72 °C for 1 minute and the PCR samples were held at 4 °C. Successful amplification was visualized on a 1% agarose gel stained with ethidium bromide and final DNA concentration determined with BioDrop µLite (BioDrop, UK).

#### 2.3.2 Barcoding and library preparation for sequencing

Following PCR amplification, DNA barcoding and library preparation were performed using the Native Barcoding Kit 96 V14 (SQK-NBD114.96, Oxford Nanopore Technologies (ONT), UK) according to the manufacturer’s protocol. For end-repair and dA-tailing, the NEBNext Ultra II module (ONT, UK) was employed with 11.5 µL of amplified DNA at a final concentration of 200 fmol. The reaction was incubated at 20 °C for 5 minutes, followed by 65 °C for 5 minutes, using a 2720 Thermal Cycler (Applied Biosystems, USA). Each sample was assigned a distinct barcode and subsequently pooled. Adapter ligation and clean-up were performed using the NEBNext Quick Ligation Module (ONT, UK) as per the manufacturer’s guidelines. DNA fragments within the 1500–1900 bp range were selected using the long fragment buffer. The final library concentration was quantified using a BioDrop µLite spectrophotometer (BioDrop, UK) and adjusted to 12 µL (50 fmol) for sequencing. The prepared sequencing library, along with the Flow Cell Priming Kit (ONT, UK), was loaded onto an R10.4.1 flow cell (ONT, UK). Sequencing was conducted on the MinION Mk1B platform using MinKNOW software (Version 24.02.6). Basecalling was performed with Dorado (Version 7.2.0) using the super-accurate basecalling model, with barcode trimming enabled at both ends and in the middle.

#### 2.3.3 Processing of amplicon sequencing for downstream analysis

The quality of the sequencing reads was assessed and visualized using Nanoplot (Version 1.42.0; de Coster et al., 2018). Following quality assessment, the data was quality filtered using MOTHUR (Version 1.48.0; Schloss et al., 2009; Winand et al., 2019; Akaçin et al., 2023). Initially, quality filtering was applied to remove primers, sequences with ambiguous bases, homopolymers longer than 8 bp, and those outside the correct size range (1000 bp to 1900 bp) or with a quality score below 25. Chimeric sequences were identified and removed using VSEARCH (Version 2.16.0; Rognes et al., 2016). Following filtering and trimming, the remaining sequences were clustered into OTUs and classified against two reference databases: SILVA Version 132 (http://www.arb-silva.de/) and EzBioCloud (https://www.ezbiocloud.net/) with a cut-off value of 80 (Quast et al., 2013; Yoon et al., 2017; Chalita et al., 2024). Sequences classified as unknown, eukaryotes, mitochondria, or chloroplasts were removed before further analysis. The raw sequence data was submitted to GENBANK as FASTQ files under accession number PRJNA1207931.

### 2.4 Data analysis

All data analyses (Secondary and Nama Karoo) were conducted with R (Version 2024.04.2; R Core Team, 2024). Before any statistical analysis was conducted, data normality was assessed with the Shapiro-Wilk Test (Hmisc package; Version 5.1.3; Harrell, 2024). Following the result, all downstream analyses were adjusted accordingly. The diversity indices of Nama Karoo and relative abundances were determined with the Microeco package (Version 1.8.0; Liu et al., 2021) and visualized with ggplot2 (Version 3.5.1; Wickham and Wickham, 2016). The significance of the difference observed between the Shannon and Simpson indices was validated with the Mann-Whitney U-test. A Permutational Multivariate Analysis of Variance (PREMANOVA) was performed to determine the significance of beta-diversity between the three sampling locations with the Vegan package (Version 2.6.8; Oksanen et al., 2024). Dunns’s test (FSA package; Version 0.9.5) was employed to determine the position of the significant difference. Clustering patterns of *Acidobacteriota* SDs of Nama Karoo samples were determined using the ward. D2 method (Murtagh and Legendre, 2014). Spearman correlation tests (Hmisc package; Version 5.1.3, Harrell, 2024) were conducted to determine the correlation between soil abiotic properties and *Acidobacteriota* SDs at each location and overall. Finally, data was assessed to determine if any co-occurrence occurred between *Acidobacteriota* and other phyla with co-occurrence network analysis and Spearman’s ranks (igraph package; Version 2.0.3; Csardi and Nepusz, 2006).

For secondary data, the clustering patterns of *Acidobacteriota* SDs and the identification of significant abiotic factors (on each specific SDs and overall) were performed using the Vegan package (Version 2.6.8; Oksanen et al., 2024). A Bray-Curtis dissimilarity matrix was computed based on SDs relative abundances, and hierarchical clustering was performed using the ward. D2 method. Principal Component Analysis (PCA) was conducted, and significant abiotic factors were fitted onto the PCA ordination using envfit() command (Vegan package; Version 2.6.8; Oksanen et al., 2024). The significance of abiotic factors was assessed using PERMANOVA with 999 permutations, and multicollinearity was addressed by calculating Variance Inflation Factors (VIF). Significant abiotic factors were visualized on the PCA plot. For all statistical tests, a p< 0.05 was considered significant.

## 3 Results

### 3.1 Clustering patterns and environmental drives: secondary data

A total of 240 samples from the secondary data representing *Acidobacteriota* SDs, were included in the analysis after passing all quality checks. The relative abundance of *Acidobacteriota* SDs observed were as follows: SD 4 (39.09%), SD 3 (21.97%) SD 6 (18.12%), Unknown (15.40%), SD 1 (5.41%) and finally SD 8 (0.008%). Clustering analysis revealed distinct groupings among the SDs. SD 1 and 3 grouped in a cluster and SDs 4, 6, 8 and Unknown. While individual abiotic factors did not significantly influence the clustering patterns, they collectively explained 29.13% of the variance observed among the SDs (p< 0.001). Among the abiotic variables, pH (p< 0.001) emerged as the most influential factor, contributing the largest portion of the explained variance. Phosphorus (P) (p< 0.01) and Calcium (Ca) (p< 0.01) were also significant contributors, while potassium (K) (p< 0.05) was weakly significant. These results indicate that abiotic factors significantly shape the overall distribution and variability of *Acidobacteriota* SDs, with their cumulative contribution providing meaningful insights into the observed patterns.

The distance-based redundancy analysis (dbRDA) revealed that abiotic factors significantly influenced the distribution of certain *Acidobacteriota* SDs. SDs 1, 3, 4, and 6 displayed highly significant models (p< 0.001), indicating that abiotic factors account for a substantial portion of the observed variation. Among the SDs, SD 3 exhibited the highest proportion of variance explained by abiotic factors, with 38.61% of its variation attributed to environmental influences. In contrast, SD 8 showed a non-significant model, suggesting that abiotic factors do not play a significant role in shaping its distribution.

Specific correlations between *Acidobacteriota* SDs and abiotic factors were further assessed with Spearman’s rank test to determine the direction and strength. SD 1 exhibited a negative correlation with pH (ρ = -0.466; p = 0.0001) and a positive correlation with carbon percentage (C %) (ρ = 0.143; p = 0.03). Subdivision 3 showed a negative correlation with pH (ρ = -0.4384; p = 0.00001) and C % (ρ = 0.256; p = 0.00016). SD4 displayed a positive correlation with pH (ρ = 0.308; p = 0.0001), a weak negative correlation with P (ρ = -0.1951; p = 0.0043), and a negative correlation with C % (ρ = -0.1926; p = 0.0048). SD 6 demonstrated a moderate positive correlation with pH (ρ = 0.4792; p = 0.001) and a weak positive correlation with P (ρ = 0.1726; p = 0.0116). SD 8 showed no significant correlations with any abiotic factors, consistent with earlier findings suggesting minimal influence of abiotic factors on this SD.

### 3.2 *Acidobacteriota* in the Nama Karoo

#### 3.2.1 Physiochemical properties and enzyme activities

All soil abiotic properties and enzyme activity results with corresponding significant p-values are summarized in **Table 1**. The table also highlights where significant differences were observed between the sampling locations. A significant difference was observed for EC, total nitrate, and available phosphate. Enzyme activities that displayed significance included acid phosphatases and urease. The significant difference for EC occurred between L 1 (65.38 ± 10.93) and both L 2 (47.49 ± 5.28) and L 3 (48.18 ± 23.27). The same pattern was identified for available phosphate (**Table 1**). The significant difference observed for acid phosphatase, urease and total nitrate occurred between L1 and L2 and L2 and L3 (**Table 1**). No significant correlation was observed for pH. Increased acid and alkaline phosphatases occurred at all three locations, with low available phosphate. Microbial activity was the highest at L1, followed by L3 and L2, respectively.

**Table 1:** Soil abiotic properties and enzyme activities for different locations.

Soil properties	Location 1	Location 2	Location 3	Significance
pH	8.08 ± 0.44	8.13 ± 0.50	7.73 ± 0.31	NS
EC	65.38 ± 10.93^a^	47.49 ± 5.28^b^	48.18 ± 23.27^c^	** ab, ac
Moisture Content (%)	0.28 ± 0.01	0.29 ± 0.02	0.28 ± 0.01	NS
Acid Phosphatase (µmol/g/h)	190.26 ± 71.66^a^	139.38 ± 22.23^b^	343.71 ± 155.71^c^	*** ab, bc
Alkaline Phosphatase (µmol/g/h)	315.34 ± 128.02	371.00 ± 159.03	364.95 ± 245.70	NS
Glucosidase (µmol/g/h)	253.73 ± 118.11	206.41 ± 115.08	224.06 ± 101.72	NS
Urease (µmol/g/h)	10.69 ± 7.70^a^	11.70 ± 4.21^b^	3.58 ± 0.45^c^	*** ac, bc
Nitrate (mg/kg)	19.44 ± 24.46^a^	51.40 ± 16.82^b^	3.58 ± 2.99^c^	*** ab, bc
Available Phosphate (mg/kg)	144.47 ± 25.15^a^	61.77 ± 21.81^b^	64.20 ± 13.35^c^	*** ab, ac
Nitrogen Mineralization (mg/kg)	0.57 ± 1.21	0.37 ± 2.38	0.36 ± 1.95 a	NS
Organic Carbon (%)	0.38 ± 0.24	0.91 ± 0.74	0.57 ± 0.36	NS
Active Carbon (mg/kg)	567.89 ± 220.41	756.22 ± 180.64	562.17 ± 243.51	NS
Microbial Activity (µmol/g/h)	315.84 ± 164.76^a^	49.14 ± 25.31^b^	95.92 ± 82.18^c^	*** ab, ac

Values are means ± standard deviation (L1 n= 12, L2 n=10; L3 n=10).

A significant difference is observed at: *p< 0.05 ** p< 0.01; *** p< 0.001. NS, not significant.

Letters (a-b) indicate where significance lies.

#### 3.2.2 The diversity and distribution of *Acidobacteriota*

##### 3.2.2.1 Alpha-diversity

**Figure 2:**
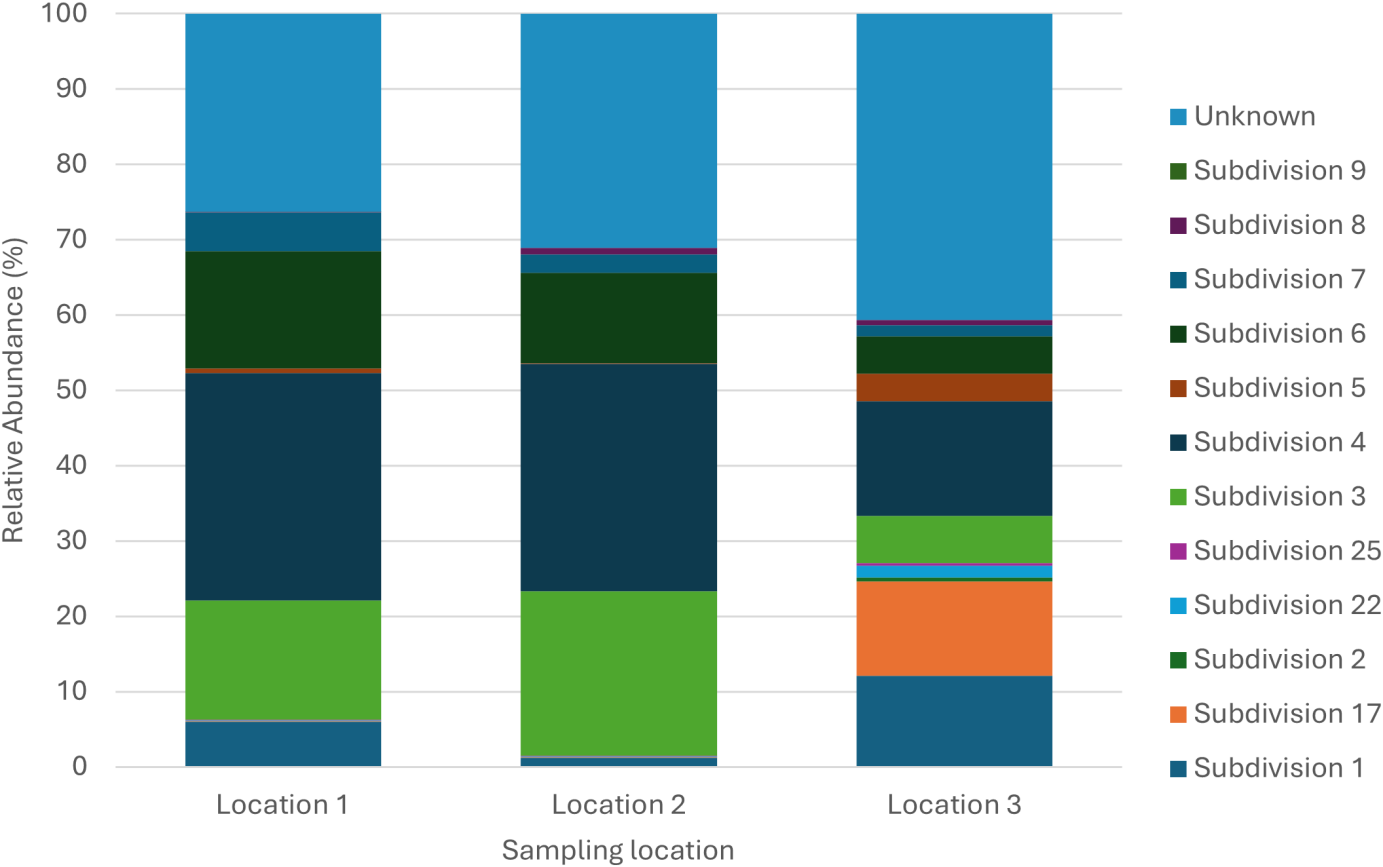
The relative abundance (%) of *Acidobacteriota* subdivisions at each sampling location.

After quality filtering and chimera removal, a total of ~1 494–941 partial 16S rRNA gene sequences were obtained, with an average amplicon length between 1000 and 1600 bp. Of these, 179–392 sequences (~12.14%) were classified as *Acidobacteriota*-affiliated reads. The relative abundance of *Acidobacteriota* ranged from 2.3% to 12.17%. There was no significant difference in the mean relative abundance of the total *Acidobacteriota* community between the three sampling locations. *Acidobacteriota* was the second most dominant phylum at L3 and the third most dominant at L1 and L2. Mean Shannon and Simpson diversity indices for *Acidobacteriota* communities are presented in **Table 2**. Overall, no significant differences were observed for these indices, indicating similar species richness, evenness, and dominance across the sampling locations. *Acidobacteriota* OTUs were classified into 28 phylotypes, representing members from various SDs (**Table 3**). **Figure 2** and **Supplementary Figures S2–S4** illustrate the major *Acidobacteriota* community composition with relative abundance ranging from 0.01% to 40.66% at the three sampling locations. The majority of the OTUs identified belong to SDs 4, 3, 6, 1 and an Unknown SD. In L1 the community composition of *Acidobacteriota* consisted of SD 4 (30.22%), Unknown (26.26%), and SD 3 (15.84%). In contrast, for both L2 and L3, Unknown (31.10%; 40.66%) accounted for the majority followed by SD 4 (30.16%; 15.21%) and SD 3 (21.83%; 6.29%). Interestingly SD 2 only occurred in L3 and SD 9 only in L1. The less frequently detected SD 25 was present at all three locations. A significant difference was observed between the relative abundances of SDs 1, 3, 4, 6, 7, 17, 22 and unknown (**Table 3**).

**Table 2:** Shannon and Simpson indices of *Acidobacteriota* at the three locations (mean ± standard deviation).

Test	Location 1	Location 2	Location 3	Significance
Shannon	4.79 ± 0.206	4.60 ± 0.201	4.55 ± 0.469	NS
Simpson	0.97 ± 0.005	0.97 ± 0.007	0.97 ± 0.008	NS

**Table 3:** The relative abundance of *Acidobacteriota* subdivisions at the three locations, (mean± standard deviation) and the significance of difference in relative abundance observed.

Subdivision	Location 1	Location 2	Location 3	Significance
Subdivision 1	5.96 ± 0.0025^a^	1.19 ± 0.001^b^	12.10 ± 0.02^c^	* ab; bc
Subdivision 2	0	0	0.52 ± 0.001	NS
Subdivision 3	15.84 ± 0.01^a^	21.83 ± 0.02^b^	6.29 ± 0.009^c^	** ac, bc
Subdivision 4	30.22 ± 0.01	30.16 ± 0.02	15.21 ± 0.01	NS
Subdivision 5	0.63 ± 0.0013^a^	0.15 ± 0.0003^b^	3.64 ± 0.01^c^	*ac; bc
Subdivision 6	15.55 ± 0.01^a^	11.98 ± 0.005^b^	5.00 ± 0.005^c^	** ac, bc
Subdivision 7	5.18 ± 0.003^a^	2.46 ± 0.001^b^	1.46 ± 0.001^c^	*** ac
Subdivision 8	0.08 ± 0.0001	0.83 ± 0.0004	0.70 ± 0.0008	NS
Subdivision 9	0.01 ± 0.00002	0	0	NS
Subdivision 17	0.13 ± 0.0001^a^	0.13 ± 0.0002^b^	12.52 ± 0.04^c^	*ac; bc
Subdivision 22	0.08 ± 0.0001	0.09 ± 0.0003	1.62 ± 0.005	NS
Subdivision 25	0.05 ± 0.00007	0.08 ± 0.0001	0.28 ± 0.0008	NS
Unknown	26.26 ± 0.01	31.10 ± 0.02	40.66 ± 0.05	NS

A significant difference is observed at: *p< 0.05 ** p< 0.01; *** p< 0.001. NS, not significant.

Letters (a-c) indicate where significance lies.

Other major taxa identified across all three sampling locations included the phyla *Pseudomonadota*, *Actinomycetota*, *Planctomycetota*, *Bacillota*, *Bacteroidota*, and *Armatimonadota*. Taxonomically described genera from SD 1 included *Acidicapsa*, *Acidipila*, *Occallatibacter*, *Candidatus* Korobacter, *Granulicella*, and *Bryocella*. Members of SD 3 included *Candidatus* Solibacter, *Bryobacter* and *Paludibaculum*. SD 4 members present were *Pyrinomonas*, *Blastocatella*, *Stenotrophobacter* and *Aridibacter*. The genera *Vicinamibacter* and *Luteitalea* were identified for SD 6 and SD 8 contained members of the class *Holophaga.*

##### 3.2.2.2 Clustering analysis

Clustering of SDs based on relative abundance at each location was assessed as well as the contribution of clustering driven by the different abiotic factors. The results in **Table 4** show the individual contributions of each abiotic factor to SD clustering. At L1, pH and available phosphate accounted for 51.14% of the total variance explained for clustering. At L2, the total clustering due to abiotic factors is 83.99%. Active carbon (24.87%), and pH (12.45%) are the strongest drivers of SD clustering at L3. At the three locations, some *Acidobacteriota* SDs consistently cluster together, while others exhibit location-specific variations. Subdivisions 25, 22, and 17 consistently group in Cluster 3 across all locations. Similarly, SDs 3, 4 and 6 consistently group in Cluster 2 at L1 and L2. However, they shift from Cluster 1 to Cluster 2 at L3, reflecting location-specific ecological variability. Subdivision 1 transitions from Cluster 1 at L1 to Cluster 3 at L2 and L3. Subdivision 8 shows a similar shift, clustering in Cluster 3 at L1 but moving to Cluster 1 at L2 and L3. The Unknown SD also shifts, grouping in Cluster 1 at L1 and L2 and transitioning to Cluster 2 at L3, but its ecological role remains uncertain due to its unidentified nature.

**Table 4:** The portion of total variance explained by abiotic factors and significance value.

Abiotic factors	*R*^2^ (%)	Significance
Location 1		
pH	38.78	***
Available Phosphate (mg/kg)	12.36	***
Active carbon (mg/kg)	5.83	***
Moisture Content (%)	2.79	**
Nitrate (mg/kg)	0.69	*
Organic Carbon (%)	0.45	*
Location 2		
pH	23.02	***
Available Phosphate (mg/kg)	60.97	***
Active carbon (mg/kg)	8.91	**
Moisture Content (%)	1.45	*
Organic Carbon (%)	4.89	**
Location 3		
pH	12.45	**
Available Phosphate (mg/kg)	47.12	***
Active carbon (mg/kg)	24.89	***
Moisture Content (%)	5.20	**
Organic Carbon (%)	9.64	*

A significant difference is observed at: *p< 0.05 ** p< 0.01; *** p< 0.001.

##### 3.2.2.3 Beta-diversity

**Figure 3:**
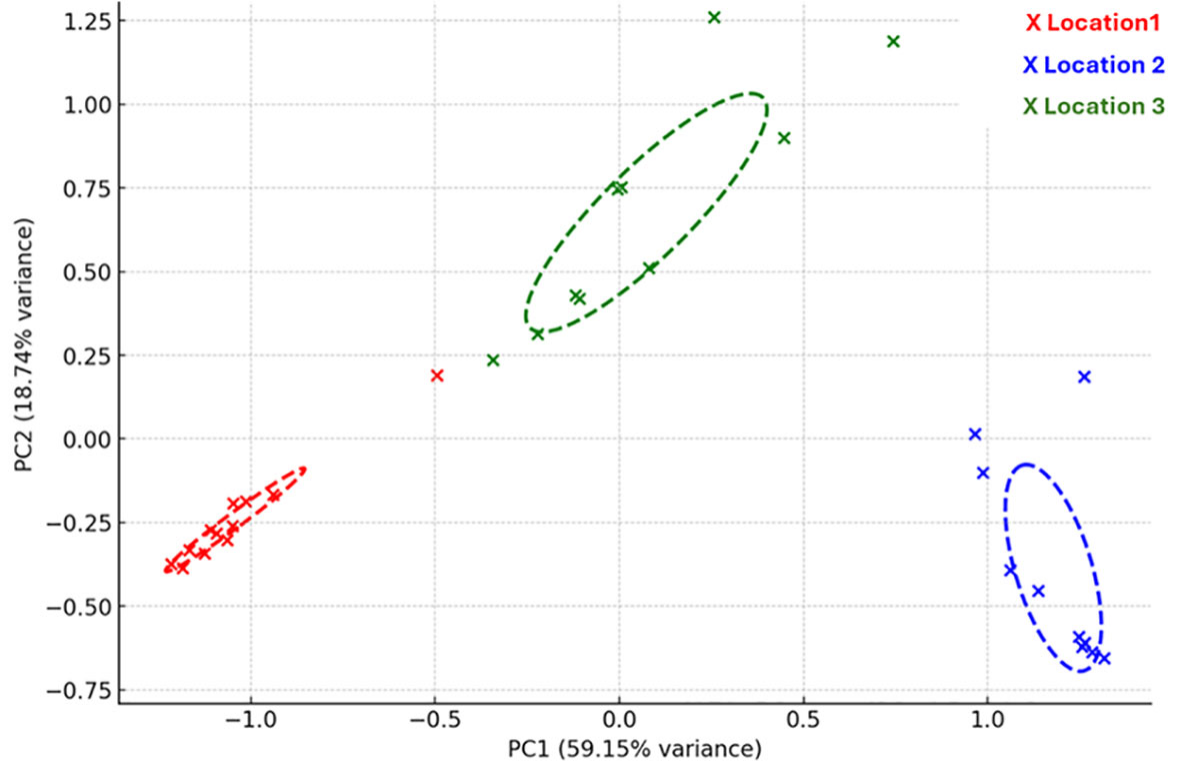
Principal Component Analysis (PCA) plot illustrating the beta-diversity (Bray-Curtis dissimilarity) across three different sites: The plot shows the distribution of samples from each site along two principal components (PC1 and PC2), explaining 59.15% and 18.74% of the variance, respectively. Colored ellipses represent 95% confidence intervals for each site, highlighting the clustering patterns of Acidobacteriota communities at the three locations.

The *Acidobacteriota* community composition between sampling locations was significantly different (p= 0.002). In **Figure 3** the PCA plot illustrates the beta-diversity (Bray-Curtis dissimilarity) across the various locations. **Supplementary Figures S5–S7** illustrate the overall variation in *Acidobacteriota* community composition in response to abiotic factors, showing how different SDs align with environmental gradients across the study locations. **Table 5** presents Spearman’s rank correlations between significant abiotic factors and SDs at specific locations.

**Table 5:** Spearman’s correlation of abiotic factors with each subdivision across sampling locations and the statistical significance.

Subdivision	Abiotic factor	Spearman ρ	Significance
Location 1			
Subdivision 4	Moisture content (%)	0.6617	*
Subdivision 4	Nitrate (mg/kg)	0.7056	**
Subdivision 6	Active carbon (mg/kg)	0.7071	**
Subdivision 8	Active carbon (mg/kg)	0.7071	**
Subdivision 3	pH	0.8374	***
Subdivision 3	Organic carbon (%)	0.6154	*
Location 2			
Subdivision 4	Organic carbon (%)	-0.7196	**
Subdivision 6	Organic carbon (%)	0.7034	*
Subdivision 8	Organic carbon (%)	-0.7363	**
Subdivision 22	pH	-0.6857	*
Subdivision 3	Nitrate (mg/kg)	0.7556	**
Location 3			
Subdivision 4	Organic carbon (%)	-0.7095	*
Subdivision 6	Available phosphate (mg/kg)	0.7391	**
Subdivision 8	Organic carbon (%)	-0.7206	**
Subdivision 22	pH	-0.7118	*
Subdivision 3	Nitrate (mg/kg)	0.7458	**
Subdivision 3	Organic carbon (%)	0.7132	*
Subdivision 7	Available phosphate (mg/kg)	0.7269	**

A significant difference is observed at: *p< 0.05 ** p< 0.01; *** p< 0.001.

The shift in the *Acidobacteriota* community composition as a whole was also influenced by soil abiotic properties. Subdivision 3 exhibited a negative correlation with acid phosphatase (p = 0.033; ρ = -0.376) and a positive correlation with nitrate (p = 0.011; ρ = 0.415). Subdivision 5 showed significant negative correlations with moisture content (p = 0.042; ρ = -0.360), urease (p = 0.018; ρ = -0.413), and nitrate (p = 0.037; ρ = -0.367). Subdivision 1 was negatively correlated with EC (p = 0.030; ρ = -0.377), while SD 2 exhibited a negative correlation with alkaline phosphatase (p = 0.048; ρ = -0.351). Subdivision 22 displayed a negative correlation with nitrate (p = 0.012; ρ = -0.437) and a positive correlation with acid phosphatase (p = 0.032; ρ = 0.379). The Unknown SD was negatively correlated with available phosphate (p = 0.044; ρ = -0.357).

The network correlation analyses revealed specific correlations between the presence of *Acidobacteriota* and other phyla. A significant positive correlation was observed for the co-occurrence between *Acidobacteriota* and *Planctomycetota* (p = 0.001; ρ = 0.531) and *Acidobacteriota* and *Armatimonadota* (p = 0.013; ρ =0.453). Members of the *Armatimonadota* included *Chthonomonas, Armatimonas* and *Fimbriimonas*. Members of *Planctomycetota* were *Pirellula, Planctopirus, Blastopirellula, Paludisphaera, Planctomicrobium, Rhodopirellula, Algisphaera, Gemmataceae, Isosphaeraceae, Pirellulaceae, Planctomycetaceae* and *Candidatus* Nostocoida.

## 4 Discussion

### 4.1 Abundance, diversity and community composition

Approximately 40% of the world’s total land surface is classified as dryland soils according to the United Nations Committee to Combat Desertification (UNCCD, 2024). Within dryland environments, arid lands, are characterized by very low precipitation per year and high levels of solar radiation (Cowan et al., 2022). The soil microbiome presence in these environments is crucial (Vásquez-Dean et al., 2020; Rodríguez et al., 2024). Earlier studies investigating microbial diversity in arid lands have reported a relative abundance of *Acidobacteriota* ranging from 0.7% to 15.4% (Scola et al., 2018; Rodríguez et al., 2024). These findings align closely with the results obtained in both the secondary data and the present study. It is important to note that all functional roles discussed are based on published genomic and physiological evidence from related *Acidobacteriota* taxa.

A recent study by Rodríguez et al. (2024) investigating the microbial impact on soil stability, formation in arid lands and the communities’ response to climate change indicated that *Acidobacteriota* was one of the most abundant and dominant taxa found, (relative abundance ~20.7%). Additionally, during the stimulated response of the community to climate change, *Acidobacteriota* abundance remained stable, reflecting their adaptation to these environments. Their association with nutrient availability, specifically nitrogen, suggests that *Acidobacteriota* may play a role in nutrient cycling and soil stabilization in these environments (Rodríguez et al., 2024). Additionally, *Acidobacteriota* exhibited a strong correlation with organic matter content, highlighting their potential contribution to carbon cycling in nutrient-poor soils (Rodríguez et al., 2024). Research on the specific SDs of *Acidobacteriota* in these environments and possible contributions of abiotic factors remains limited, with most studies concentrating on the broader phylum level. This limitation is especially evident within the South African context, where detailed analyses of SDs are scarce (Conradie and Jacobs, 2021).

The lack of significant differences in Shannon and Simpson diversity indices between the three sampling locations of the Nama Karoo suggests that the overall bacterial community diversity and evenness are relatively similar across these locations (**Table 3**). In contrast, the beta-diversity differed significantly between the three sampling locations, indicating greater dissimilarity between microbial communities (**Figure 3**). These differences reflect distinct environmental factors or conditions, such as soil pH, moisture, or nutrient availability, which influence the microbial communities at each site. The observed relationship with soil properties can be linked to the ecological traits of *Acidobacteriota* and the adaptive strategies of desert microbial communities.

#### 4.1.1 Dominant and rare subdivisions

In the Nama Karoo, as well as in secondary data analyses, SDs 4, 3, and 6 collectively dominated the Acidobacteriota community. Subdivision 4 was the most abundant and consistently stable, reflecting its ability to thrive in arid environments characterized by low moisture, slightly alkaline pH, and nutrient limitation (Wüst et al., 2016; Ivanova et al., 2020). Cultivated members of SD 4, such as Aridibacter, Blastocatella, and Pyrinomonas, demonstrate metabolic strategies that support survival under these conditions, including oligotrophic growth, utilization of diverse carbon substrates, and resistance to desiccation, collectively enabling carbon cycling, polysaccharide degradation, and soil stabilization (Huber et al., 2014; Crowe et al., 2014; Ivanova et al., 2020; Huber et al., 2017).

Similarly, SD 3 and SD 6 showed clear associations with soil pH and nutrient availability. SD 3 members are adapted to mildly acidic soils and appear capable of exploiting available nitrate and organic carbon, suggesting metabolic versatility that supports persistence in oligotrophic, arid soils (Huber and Overmann, 2018). Subdivision 6, including representatives such as Vicinamibacter silvestris, tolerates a broad pH range and utilizes diverse carbon sources, likely explaining its occurrence in arid soils with variable pH and low nutrient content (Mukherjee et al., 2014; Kielak et al., 2016; Kalam et al., 2020). The consistent clustering of these SDs further indicates shared ecological preferences, metabolic traits, and niche adaptations that underpin their dominance in arid landscapes.

Rare SDs exhibited narrower ecological niches and strong location-specific dependencies. In this study, SD 9 was exclusively present at Nama Karoo L3, highlighting its restricted habitat preference. Notably, SD 17 (Class *Vicinamibacteria*) and SD 25 consistently clustered together across all three locations, suggesting shared ecological adaptability. Consistent with these findings, *Vicinamibacteria* and SD 25 were among the most abundant classes in a previous study by Dedysh et al., 2022. Their research showed that fens were dominated by *Vicinamibacteria*, particularly members of the order *Vicinamibacteriales*, which comprised 23.1% to 58.9% of all *Acidobacteriota* sequences, alongside a significant presence of uncultured SD 17. This consistent clustering pattern further suggests functional similarities and ecological overlap between SD 17 and SD 25, reinforcing their adaptability across different environments. Additionally, SD 2 was exclusively found at L3, further emphasizing its potential niche specialization and highlighting the role of location-specific factors in shaping the distribution of rare SDs.

### 4.2 Key abiotic drivers at each location of Nama Karoo

Previous research indicates that SD 4 preferentially occurs in soils with low moisture and low carbon availability (Kalam et al., 2020; Eichorst et al., 2011), suggesting adaptations to resource-limited, arid environments. Several described SD 4 members, such as *Aridibacter famidurans* and *Aridibacter kavangonensis*, have been isolated from arid savannah soils, supporting their ecological specialization for these conditions. This preference is consistent with the oligotrophic lifestyle of SD 4 members, which enables efficient utilization of scarce carbon sources. Some members may also possess pathways for carbon dioxide fixation (Bryant et al., 2007), reducing dependence on external organic carbon. SD 4 representatives are generally slow-growing, aerobic chemoorganoheterotrophs capable of metabolizing a wide range of carbon substrates (Pascual et al., 2015), providing the physiological flexibility required to survive in low-nutrient soils. The positive correlation of SD 4 with nitrate is consistent with the metabolic capabilities of cultivated members, such as *Aridibacter nitratireducens*, which can utilize nitrate as an electron acceptor and metabolize a variety of organic substrates (Huber et al., 2017). While these organisms are generally adapted to nitrogen-limited environments, their ability to exploit available nitrate allows them to persist and maintain activity when nitrate becomes locally abundant.

Subdivision 6 displayed a positive correlation with both active and organic carbon, suggesting a functional role in soil carbon cycling. While direct experimental studies on SD 6 are limited, genomic analyses of cultivated and closely related *Acidobacteriota* indicate the presence of diverse carbohydrate-active enzymes and pathways for utilizing complex carbon compounds (Kielak et al., 2016; Kalam et al., 2020). These metabolic traits suggest that SD 6 members are capable of decomposing both labile and more recalcitrant organic matter, which could explain their higher abundance in carbon-rich soils. Such substrate versatility and potential for carbon degradation provide a mechanistic basis for the observed correlations, extending our understanding of SD 6 ecological function. These findings highlight the need for further research to experimentally confirm the metabolic pathways and carbon utilization strategies of SD 6.

Subdivision 8 had a positive correlation with active carbon and a negative correlation with organic carbon. This also represents a novel contribution to the understanding of this SD. The positive correlation with active carbon suggests that SD 8 may rely on the readily accessible pool of soil organic carbon for its metabolic activities. Active carbon, strongly associated with microbial activity and nutrient cycling, indicates that SD 8 could play a role in the rapid turnover of carbon in soils, utilizing energy-rich substrates during periods of high microbial activity (Yang et al., 2024). In contrast, the negative correlation with organic carbon implies that SD 8 may avoid soils with high stable organic matter content, potentially due to competitive disadvantages. This suggests that SD 8 thrives in soils where short-term carbon availability (active carbon) is prioritized over long-term nutrient reservoirs (organic carbon).

Subdivision 3 displayed a positive correlation with pH, consistent with previous findings demonstrating that its members tolerate a broad pH range (Jones et al., 2009; Ivanova et al., 2020). In contrast, other studies have reported a negative correlation between SD 3 abundance and pH (Foesel et al., 2013; Dedysh et al., 2017), indicating substantial physiological and ecological variability within this subdivision. This apparent inconsistency suggests that SD 3 comprises metabolically diverse and potentially undescribed taxa with flexible pH tolerance mechanisms, enabling persistence across heterogeneous soil environments. Such physiological plasticity represents an adaptive advantage in arid soils, where pH can vary spatially and temporally due to low buffering capacity and episodic moisture inputs.

Nitrate is a key nitrogen source for many soil microorganisms, and the positive correlation observed between SD 3 and nitrate concentrations provides insight into its potential ecological strategies. Although SD 3 members are commonly associated with oligotrophic environments, this relationship suggests that certain taxa within this subdivision may retain the capacity to utilize nitrate when conditions permit. This is supported by physiological evidence from cultivated representatives such as *Bryobacter aggregatus*, which can assimilate nitrate as a nitrogen source (Kulichevskaya et al., 2010). Such metabolic flexibility may allow SD 3 members to exploit transient increases in nitrogen availability following rainfall or mineralization events, a common feature of arid ecosystems.

Additionally, the positive correlation between SD 3 and organic carbon suggests a role in the degradation of more stable organic compounds, contributing to the gradual release of bioavailable carbon in nutrient-poor soils. This capability is consistent with previous reports describing *Acidobacteriota* involvement in the breakdown of complex organic matter (Rawat et al., 2012). Together, broad pH tolerance, conditional nitrate utilization, and the capacity to process stable organic carbon provide a mechanistic framework explaining the widespread distribution and persistence of SD 3 in arid and oligotrophic soil environments.

Limited information is available on SD 22, however, its negative correlation with pH suggests that, like SD 1, it thrives in lower pH environments. Similarly, SD 7’s positive correlation with available phosphate points to a potential ecological role. However, more studies are required to confirm these findings. Due to the scarcity of cultivated representatives and functional analyses, no definitive conclusions can be drawn, highlighting the need for further research.

### 4.3 Abiotic drivers of *Acidobacteriota* subdivisions distribution

#### 4.3.1 Nama Karoo insights

Subdivision 3 members are commonly found in mildly acidic soils, consistent with their physiological tolerance for a broader pH range (Jones et al., 2009; Ivanova et al., 2020). The observed negative correlation with acid phosphatase activity suggests that SD 3 may not rely on phosphatase-mediated phosphorus acquisition. Instead, these organisms may obtain phosphorus indirectly through microbial interactions, such as cross-feeding, where other microbes release phosphate as a metabolic by-product that SD 3 can utilize (Deng et al., 2022; Jara-Servin et al., 2023). Additionally, the mildly acidic soils preferred by SD 3 may naturally contain more bioavailable phosphorus, further reducing the necessity for acid phosphatases (Kielak et al., 2016; Kalam et al., 2020). In contrast, soils with high acid phosphatase activity may indicate phosphorus limitation, favoring microbes capable of producing these enzymes, whereas SD 3 thrives in less phosphorus-limited conditions.

The positive correlation of SD 3 with nitrate suggests that some members may exploit nitrate as a nitrogen source, consistent with known capabilities of taxa such as Bryobacter aggregatus (Kulichevskaya et al., 2010). Taken together, these patterns indicate that SD 3’s distribution is shaped by a combination of metabolic traits, nutrient acquisition strategies, and interactions with co-occurring microbes, providing a mechanistic explanation for its ecological niche in mildly acidic, moderately nutrient-rich soils.

Subdivision 5 is one of the lesser-known *Acidobacteriota* subdivisions, with limited information available due to the absence of described cultivated members. To our knowledge, this study represents the first report of SD 5 in arid regions of South Africa. Microbial communities in arid environments commonly employ survival strategies such as dormancy, slow growth, or biofilm formation to persist during prolonged dry periods (Huber et al., 2022a). The observed negative correlation between moisture and SD 5 suggests an adaptation to drier conditions, where reduced water availability may limit competition for resources and favor organisms capable of maintaining metabolic activity under stress (Huber et al., 2022b). These organisms may therefore persist or increase in relative abundance during dry periods when moisture-dependent taxa are less active, potentially relying on low-energy, oligotrophic metabolic strategies to maintain viability under nutrient- and water-limited conditions (Flieder et al., 2021; Kristensen et al., 2021; Cao et al., 2022; Huber et al., 2022a). The negative correlation between SD 5 and both nitrate concentrations and urease activity further supports a mechanistic interpretation based on metabolic efficiency. Genomic and physiological studies indicate that not all *Acidobacteriota* subdivisions utilize nitrate as an electron acceptor, instead relying on low-energy, oligotrophic metabolic strategies that minimize energetic costs under nutrient-limited conditions (Flieder et al., 2021; Kristensen et al., 2021; Cao et al., 2022; Kato et al., 2022). Such energy-efficient metabolic configurations are advantageous in arid soils, where nitrogen availability is limited, spatially heterogeneous, and temporally variable. The association of SD 5 with low-nitrogen environments is further supported by its negative correlation with high urease activity, which often reflects intensified nitrogen mineralization in nitrogen-limited soils (Roscoe et al., 2000).

Subdivision 1 displayed a significant negative correlation with EC, indicating a preference for soils with lower salinity. Although studies directly linking SD 1 abundance to soil EC are limited, physiological evidence from cultivated representatives provides mechanistic insight into this pattern. Several described members of SD 1, including *Silvibacterium dinghuense*, *Acidobacterium ailaaui*, *Acidicapsa ligni*, and *Silvibacterium bohemicum*, exhibit limited tolerance to NaCl concentrations above 0–2.5%, reflecting sensitivity to osmotic and ionic stress (Kulichevskaya et al., 2012; Lladó et al., 2016; Myers and King, 2016; Zhang et al., 2022). This physiological constraint suggests that SD 1 lacks specialized salt-adaptation mechanisms, such as efficient osmolyte accumulation or ion extrusion systems, thereby favoring its persistence in low-salinity arid soils rather than saline environments.

Subdivision 2 exhibited a negative correlation with high alkaline phosphatase activity, suggesting that SD 2 may not directly contribute to phosphatase production. This pattern implies that other organisms are primarily responsible for phosphorus mineralization in these soils. Alternatively, SD 2 could participate indirectly in phosphorus cycling through mutualistic or commensal interactions with microbes that produce or utilize alkaline phosphatase, allowing it to access released phosphate without expending energy on enzyme production (Bakki et al., 2024). Its lower abundance in soils with high alkaline phosphatase activity may indicate competitive exclusion, where SD 2 is less adapted to environments dominated by fast-growing, phosphatase-producing taxa. Overall, these patterns suggest that SD 2 occupies a niche strategy focused on indirect nutrient acquisition, highlighting the importance of microbial interactions in shaping its ecological distribution.

The only described member from SD 22 has been isolated from marine sediments where its role in sulphur cycling was reported (Flieder et al., 2021). Therefore, the detection of this group in these soil environments was surprising. However other members of this phylum have been identified in both aquatic and terrestrial environments (Kishimoto et al., 1991; Barns et al., 1999).

#### 4.3.2 Secondary data insights

Earlier research has consistently shown that both SDs 1 and 3 thrive in slightly acidic environments. The negative correlation observed in the secondary data further supports this pattern, reinforcing the association between SD 1 and SD 3 abundance and lower soil pH (Jones et al., 2009; Lauber et al., 2009; Kielak et al., 2016; Dedysh et al., 2017; Dedysh and Sinninghe Damsté, 2018; Ivanova et al., 2020; Kalam et al., 2020; Conradie and Jacobs, 2021). Their positive correlation with total carbon aligns with the metabolic versatility reported in related *Acidobacteriota*, which possess the genomic potential to degrade a range of organic compounds, including complex carbon substrates (Kielak et al., 2016; Kalam et al., 2020). This suggests that SDs 1 and 3 could contribute to soil carbon cycling by utilizing both readily available and more recalcitrant carbon sources. The consistent clustering of SDs 1 and 3 indicates shared ecological preferences, potential overlap in metabolic traits, and similar niche adaptations. While direct experimental data on SD 1 and SD 3 remain limited, these patterns collectively suggest that their distribution is influenced by pH tolerance, carbon utilization strategies inferred from related taxa, and niche complementarity, highlighting directions for future functional research.

Subdivision 4 is frequently associated with arid lands as previously mentioned, where nutrient levels, including phosphorus, are often low (Huber et al., 2014). Its ability to survive in higher pH could be explained, as currently described members of this SD can tolerate a range from 4 to 10, with optimum at between 7 and 9 (Huber et al., 2014). The negative correlation with both P and C % highlights its ability to compete effectively in oligotrophic environments with limited phosphorus resources.

The first cultured representative of SD 6, *Vicinamibacter silvestris*, was found to grow within a pH range of 4.7 to 9.0, demonstrating adaptability to diverse soil pH conditions (Mukherjee et al., 2014). This broad pH tolerance likely explains its presence in arid soils, where pH can vary due to low organic matter content and mineral heterogeneity (Maestre et al., 2015; Tidimalo et al., 2024). SD 6 also displays a positive correlation with both active and total organic carbon, suggesting a capacity to utilize a range of carbon substrates, which would further support its survival in nutrient-limited arid environments (Kielak et al., 2016; Kalam et al., 2020). While research on SD 6 and soil phosphorus interactions is limited, its ability to tolerate variable pH and utilize diverse carbon sources likely underpins its ecological success in arid regions. Further studies are required to experimentally confirm these functional traits.

In contrast, SD 8 showed no significant correlations with any abiotic factors. This SD may exhibit broad tolerance or versatility, allowing it to adapt to a wide range of environmental conditions. This adaptability suggests that its abundance and distribution are not strongly influenced by specific abiotic factors such as pH, nutrients, or moisture. Instead, SD 8 might rely more on biotic interactions, including relationships with other microorganisms to shape its ecological role and distribution. Additionally, SD 8 may perform generalist roles in soil ecosystems, enabling it to function effectively across diverse environments without dependence on specific abiotic conditions (von Meijenfeldt et al., 2023). This functional redundancy could explain its even distribution and lack of strong associations with abiotic factors.

A large portion of the *Acidobacteriota* found in secondary data and the current study remained unknown due to challenges in cultivation, slow growth, and the limited resolution provided by culture-independent techniques. Advances in genomic tools and improved culturing methods may help uncover more information about these elusive bacteria in the future. This observation is not a new phenomenon as other studies have reported the same limitation (Conradie and Jacobs, 2021; Coluccia and Besaury, 2023).

It is important to note that in this study, Oxford Nanopore Technologies was used in this study, offering significant advantages due to its ability to generate longer read lengths and capture greater variability by targeting the entire V1–V9 region of the 16S rRNA gene. This approach enabled the detection of a broader spectrum of bacterial diversity, including elusive groups such as SD 9, which were previously underrepresented. Compared to earlier sequencing methods that focused on shorter regions, ONT sequencing provided superior resolution, allowing differentiation between closely related SDs. The improved resolution is evident in the detection of SDs in this study compared to secondary data, where shorter read lengths likely led to the underrepresentation of certain groups. Numerous studies have also demonstrated ONT’s ability to enhance microbial diversity detection in both mock communities and environmental samples (Szoboszlay et al., 2023; Waechter et al., 2023), reinforcing its value in microbial ecology research.

### 4.4 *Acidobacteriota* and other phyla

The co-occurrence observed for bacteria in a microbial community could be due to shared environmental preferences, syntropy, nutrient cycling, metabolic redundancy, and protection among others (Røder et al., 2018; Chen et al., 2022; Kajihara and Hynson, 2024). The co-occurrence of *Acidobacteriota* with *Planctomycetota* and *Armatimonadota* in various soil environments can be attributed to their shared ecological roles and preferences for certain environments (Vitorino and Lage, 2022; Coluccia and Besaury, 2023; Cui et al., 2023; Øvreås et al., 2025). Studies have identified that both *Acidobacteriota* and *Planctomycetota* are well-adapted to environments with limited nutrient availability due to their ability to degrade complex organic matter (Klimek et al., 2024). Several genes responsible for complex carbohydrate degradation have been found in *Acidobacteriota* and *Planctomycetota* (Kielak et al., 2016; Kalam et al., 2020; Coluccia and Besaury, 2023). This environmental preference extends to *Acidobacteriota* and members of the Armatimonadota, genus *Chthonomonas*, both of which have been identified in geothermal soils. In these environments, both groups play essential roles in the decomposition of polysaccharides (Crowe et al., 2014; Lee et al., 2014).

A study evaluating the ecological roles of uncultivated bacteria with long-read metagenomics revealed that *Acidobacteriota* and *Armatimonadota* can co-occur due to metabolic interdependence in nitrogen cycling, as it became evident that *Acidobacteriota* is responsible for NO reduction to N_2_O, and *Armatimonadota* for N_2_O reduction to N_2_, respectively (Kato et al., 2022). This process might also hold true for arid lands. *Acidobacteriota* and *Armatimonadota* have genes for hydrogenases involved in the production and oxidation of H_2_ indicating possible functional redundancy as both could perform overlapping functions related to the hydrogen cycle (Kato et al., 2022).

The co-occurrence of *Acidobacteriota* with *Planctomycetota* and *Armatimonadota* may be attributed to the physical protection offered by these groups. Both *Planctomycetota* and *Armatimonadota* are known to form biofilms or aggregates (Bengtsson and Øvreås, 2010; Carlton et al., 2023; Liew et al., 2024). This association could shield *Acidobacteriota* from environmental stressors such as dehydration, radiation, or fluctuating temperatures (Zhao et al., 2023; Dhiman et al., 2024; Ragupathi et al., 2024). Moreover, the exopolysaccharides produced by *Planctomycetota* and *Armatimonadota* within these biofilms may help retain moisture, providing further benefits to *Acidobacteriota*, particularly in arid or saline environments.

## 5 Conclusion and final remarks

Both secondary data and Nama Karoo samples provide valuable insights into the diversity, ecological roles, and environmental adaptations of *Acidobacteriota* in arid regions. Their diversity and interactions with soil properties highlight their ecological flexibility, particularly their ability to thrive under low water and nutrient availability. The dominance of SD 4, SD 3, and SD 6 suggests their central role in these environments: Subdivision 4 thrives in low-carbon, low-moisture soils due to its oligotrophic lifestyle and potential carbon dioxide fixation; SD 3 correlates with organic carbon and nitrate, indicating a role in organic matter decomposition; and SD 6 is linked to carbon cycling through its association with active and organic carbon. Rare SDs showed site-specific patterns, with SD 9 and SD 2 restricted to L3, while SD 17 and SD 25 consistently clustered together, suggesting shared ecological niches. Soil properties strongly influenced distributions: SDs 1 and 3 were associated with lower pH, while SDs 4 and 6 favored alkaline soils. Subdivision 5’s negative correlation with moisture suggests adaptation to arid conditions, whereas SD 7’s positive correlation with phosphate points to a role in phosphorus cycling.

Microbial interactions also shaped distributions, with *Acidobacteriota* co-occurring with *Planctomycetota* and *Armatimonadota* in carbohydrate degradation and nitrogen cycling, where functional interdependencies likely include NO reduction by *Acidobacteriota* and N_2_O reduction by *Armatimonadota*. Biofilm formation by these partners may further support *Acidobacteriota* survival under stress.

*Acidobacteriota* exhibit extraordinary versatility, yet they remain highly environment-specific, making them possible specialists within their ecosystems. This specialization likely contributes to the difficulty in culturing them, as their growth requirements may be tightly linked to specific environmental conditions that are difficult to reproduce in the laboratory. It is also important to note that soil sampling provides a limited overview of microbial communities at a single point in time. Microbial populations are dynamic, shaped by environmental fluctuations, seasonal changes, and microbial interactions. Furthermore, while this study examined several abiotic factors, many remain unexplored. Biotic interactions such as microbial competition, cross-feeding, and symbioses are likely to play significant roles but remain poorly understood. The clustering of certain SDs suggests potential functional redundancy, where different groups may perform overlapping roles in the ecosystem. This complexity underscores the importance of considering a wider range of factors when interpreting microbial patterns. For example, the secondary data only assessed total carbon, whereas the Nama Karoo samples incorporated active and organic carbon, which provided more meaningful ecological insights and demonstrated how different carbon pools shape microbial distributions.

Future research should build on this by incorporating additional environmental parameters, applying metagenomic and transcriptomic approaches, and prioritizing the characterization of metabolic pathways of dominant SDs. Testing the resilience of these groups under changing conditions and focusing on poorly understood SDs such as SD 5, SD 22, and SD 8 will be particularly valuable. Finally, this study advances the understanding of *Acidobacteriota* in arid soils of Southern Africa by the incorporation of secondary and newly generated data, highlighting their importance in nutrient cycling, soil stability, and microbial networks, while pointing to key knowledge gaps requiring genomic and cultivation-based approaches.

## Data Availability

The datasets presented in this study can be found in online repositories. The names of the repository/repositories and accession number(s) can be found here: https://www.ncbi.nlm.nih.gov/bioproject/PRJNA1207931/.
