# Supplementary material for: The prevalence and distribution of Acidobacteriota in the Nama Karoo of South Africa
Source: Front Microbiomes. 2026 Feb 13;5:1653994. doi: 10.3389/frmbi.2026.1653994 (PMC12993689; doi:10.3389/frmbi.2026.1653994)
Supplement: Supplementary file 1 [file SupplementaryFile1.docx]

**Supplementary information**

**Figure S1:** Visual representation of sampling sites from previously published studies on microbial diversity in arid regions incorporated into the present analysis. Each yellow dot denotes a specific sampling location within the secondary dataset.

**Figure S2**: Relative abundance (%) of different Acidobacteriota subdivisions in each sample at Location 1.

**Figure S3**: Relative abundance (%) of different Acidobacteriota subdivisions in each sample at Location 2.

**Figure S4**: Relative abundance (%) of different Acidobacteriota subdivisions in each sample at Location 3.

**Figure S5 :** Canonical analysis of principal coordinates (CAP) plot illustrating the clustering of Acidobacteriota subdivisions based on abiotic factor loadings at Location 1. CAP1 (84.68%) explains the majority of the constrained variance, with subdivisions positively associated with active carbon and available phosphate aligning to the right, while subdivisions negatively associated with low pH, organic carbon, and moisture content align to the left. CAP2 (9.21%) highlights additional variability influenced by secondary factors. Red arrows represent abiotic factor loadings and their directional influence on subdivisions

**Figure S6** : Canonical analysis of principal coordinates (CAP) plot showing subdivision clustering in relation to abiotic factors at Location 2. CAP1 (39.47%) captures the primary variance, driven by gradients in active carbon and available phosphate (positive axis) versus pH and organic carbon (negative axis). CAP2 (20.42%) reflects secondary variability associated with moisture content and nitrate. Red arrows indicate the direction and strength of abiotic factor influence

**Figure S7**: Canonical analysis of principal coordinates (CAP) plot depicting the clustering of Acidobacteriota subdivisions at Location 3, with CAP1 (41.5%) and CAP2 (23.6%) collectively explaining the variance in subdivision distribution. Subdivisions positively aligned with CAP1 are strongly influenced by active carbon and available phosphate, while negatively aligned subdivisions are linked to low pH and organic carbon. CAP2 reflects additional variation linked to moisture content. Red arrows indicate the magnitude and direction of abiotic factor influence on subdivisions.
